# Analysis of gene expression in operons of *Streptomyces coelicolor*

**DOI:** 10.1186/gb-2006-7-6-r46

**Published:** 2006-06-02

**Authors:** Emma Laing, Vassilis Mersinias, Colin P Smith, Simon J Hubbard

**Affiliations:** 1 Faculty of Life Sciences, The University of Manchester, Manchester M13 9PT, UK; 2 Current Address: School of Biomedical and Molecular Sciences, University of Surrey, Guildford GU2 7XH, UK; 3 Functional Genomics Laboratory, School of Biomedical and Molecular Sciences, University of Surrey, Guildford GU2 7XH, UK

## Abstract

**Background:**

Recent studies have shown that microarray-derived gene-expression data are useful for operon prediction. However, it is apparent that genes within an operon do not conform to the simple notion that they have equal levels of expression.

**Results:**

To investigate the relative transcript levels of intra-operonic genes, we have used a Z-score approach to normalize the expression levels of all genes within an operon to expression of the first gene of that operon. Here we demonstrate that there is a general downward trend in expression from the first to the last gene in *Streptomyces coelicolor *operons, in contrast to what we observe in *Escherichia coli*. Combining transcription-factor binding-site prediction with the identification of operonic genes that exhibited higher transcript levels than the first gene of the same operon enabled the discovery of putative internal promoters. The presence of transcription terminators and abundance of putative transcriptional control sequences in *S. coelicolor *operons are also described.

**Conclusion:**

Here we have demonstrated a polarity of expression in operons of *S. coelicolor *not seen in *E. coli*, bringing caution to those that apply operon prediction strategies based on *E. coli *'equal-expression' to divergent species. We speculate that this general difference in transcription behavior could reflect the contrasting lifestyles of the two organisms and, in the case of *Streptomyces*, might also be influenced by its high G+C content genome. Identification of putative internal promoters, previously thought to cause problems in operon prediction strategies, has also been enabled.

## Abstract 2

Analysis of the relative transcript levels of intra-operonic genes in *Streptomyces coelicolor *suggests significant levels of internal regulation.

## Background

The analysis of gene expression patterns observed over a range of conditions and time points has become widely used in modern biology to discover relationships between different genes in a genome. This can involve clustering genes into co-expressed sets to try and predict common functions and regulatory relationships, or to determine differential expression in different conditions to provide insight into the function of specific genes. Additionally, in prokaryotic organisms, the relationships inferred from gene co-expression should also provide clues to the organization of genes into operons and regulons. Since operons are, by definition, a transcriptional unit containing genes that are co-regulated as a single polycistronic message, they are, therefore, deemed to be functionally similar. Hence, an understanding of operon structure and regulation forms a basis on which to build regulatory networks.

Given the importance of operons to prokaryotic gene function and regulation, several approaches have been developed to try and predict them, exploiting genome sequences and other related features. As operon structure has been observed to be relatively poorly conserved [1-4] non-homology based prediction of operons has predominated. These methods use the basic principles that genes within the same operon are controlled by a single promoter, tend to be close together [5], terminate at a single transcription terminator, and are transcribed at similar levels. Several groups have developed computational methods to predict operons that adopt these principles, either through the use of sequence information alone [6] or by combining it with microarray data [7,8] and/or by including functional annotation [5,9-11] in *E*s*cherichia coli *or *Bacillus subtilis*. The use of microarray data and/or functional data improves the quality of operon prediction above that achieved from sequence alone and in addition offers some experimental validation of the predictions [7,8,11].

Through the presence of a promoter(s) and other regulatory features such as *cis*-acting transcription factor-binding sites upstream of the first gene of an operon, it is generally assumed that genes within an operon are expressed at equal levels. Typically, this equal expression is measured through distance metrics such as Pearson correlation or Euclidean distance where a score of 1 or 0 (respectively) is considered to be more likely with operonic pairs than non-operonic pairs. Indeed, recent studies in *E. coli *[8] and *B. subtilis *[7] have shown that this is the case, with operonic pairs of genes showing high correlation in gene expression using either metric. However, although microarray data are useful in predicting operons, the ideal condition of perfectly correlated gene expression, even within well characterized operons, is not observed experimentally, and the distinction between operonic and non-operonic pairs is not straightforward.

In the light of these findings we were motivated to analyze the patterns of expression across documented operons in *Streptomyces coelicolor*, an actinomycete with a high G+C content genome that is responsible for the production of about two-thirds of all natural antibiotics currently available, and to compare them with our knowledge of known *E. coli *operons. This was driven from an interest in *S. coelicolor *itself, which is a complex bacterium equipped with an unusually large number of transcription factors, including 65 sigma factors [12], and also to provide a third, phylogenetically diverse, bacterial species in which to examine operon-expression relationships. Furthermore, we use the determined pattern of expression across operons to identify potential internal *cis*-acting control sites by combining microarray-derived expression profiles with transcription factor binding site (TFBSs) and terminator prediction algorithms. This study reveals that the control of gene expression in operons in *Streptomyces *differs from, and is more complex than that observed in *E. coli *and *B. subtilis*, and is likely to demonstrate more internal control.

## Results and discussion

**Figure 1:**
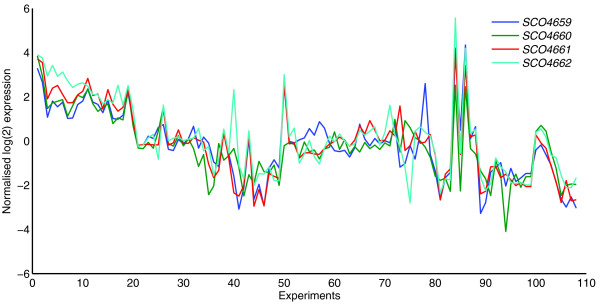
Example operon expression profile. Expression profile across all 107 experiments for all genes within the *rspL-tuf1 *operon in *S. coelicolor*.

In order to compare expression levels of genes within operons, we considered several metrics, concluding that the Pearson correlation coefficient provides a superior measure of the direction or change in gene expression over a set of experiments. This has also been observed by other workers [7] and suggests that the gene expression profiles of operonic members are co-ordinated (that is, go up and down in a correlated fashion) but not necessarily in terms of absolute expression level. This idea is illustrated in Figure 1, which depicts the normalized expression profile of 107 experiments for each of the four genes found within the known *S. coelicolor rspL*-*tuf1 *operon [13]. Figure 1 illustrates cases where genes in the same operon have similar trajectory patterns over a variety of experiments, but varying expression levels for individual experiments.

**Figure 2:**
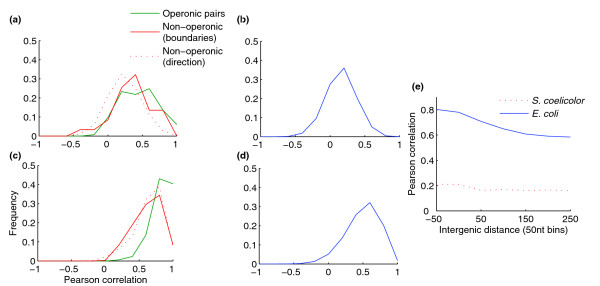
Correlations in intra-operonic and non-operonic gene expression. Correlations in intra-operonic and non-operonic gene expression in **(a) ***S. coelicolor *and **(c) ***E. coli*. Random gene pair correlation distribution (after 10,000 simulations) in **(b) ***S. coelicolor *and **(d) ***E. coli*. **(e) **Shows upstream intergenic distance versus Pearson correlation distributions for *S. coelicolor *and *E. coli*.

**Figure 3:**
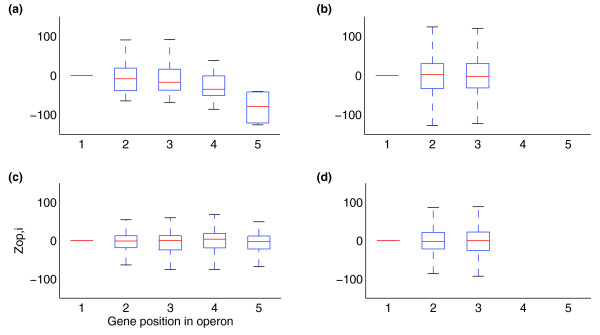
Variation in generalised operon gene expression. Box plot diagrams for all *Z*_*op*,*i *_values calculated for genes at position *k *in **(a) **all known *S. coelicolor *operons, **(b) **adjacent genes known not to be in an operon in *S. coelicolor*, **(c) **all known *E. coli *operons, and **(d) **adjacent genes known not be in an operon in *E. coli*.

When the normalized expression levels of genes within operons are compared in *S. coelicolor *and *E. coli *(Figure 2a,c), the intra-operonic pairs show a higher degree of correlation than those not in operons, using either of our definitions for non-operonic (either through direction, or crossing a true operon boundary). For example, the mean correlation values in *S. coelicolor *are 0.34, 0.26, 0.14 for known operons, non-operons (via direction), and non-operons (via boundaries), respectively. The equivalent values in *E. coli *are 0.74, 0.53, 0,54. However, the trend is more marked between known operonic gene pairs and randomly selected gene pairs (*p* < 0.01 after *t *test; Figure 2b,d). Interestingly, the most significant trend is noted between random gene pairs in *E. coli *and *S. coelicolor*, where randomly selected gene pairs are significantly (*p* < 0.01 after *t *test) more highly correlated than with mean correlation coefficients of 0.4 and 0.06, respectively (Figure 2b,d). This is also backed up by Figure 2e, which shows that the closer gene pairs are together (using adjacent gene pairs on the same strand in the genome), the stronger the correlation in their expression profiles, independent of their operonic status. Again, the similarity in gene expression between adjacent genes transcribed in the same direction is much larger in *E. coli *compared to *S. coelicolor *(Figure 3e). This has important implications for operon prediction methods that use microarray data directly or to validate predictions [7,8,11,14] because, particularly in *E. coli*, gene proximity is highly correlated with co-expression regardless of whether the genes are members of the same operon.

Taken together, these results suggest that control of genes within characterized operons in *S. coelicolor *is more complex, and that the regulation of expression at adjacent loci is more diverse than in *E. coli*. Indeed, the large differences in gene expression patterns observed between the two species were unexpected and we tested for bias in the data sets that might cause this. However, we found no systematic differences in the number and type of experiments (time-course derived data and/or single perturbations) or absence of variation of individual gene expression (measured by an entropy value, (E Laing and S Hubbard, unpublished data)) that would lead to higher correlations. Indeed, the latter entropy calculations suggested there is more variation in the *E. coli *data sets. The apparent increased complexity of genetic control in *S. coelicolor *might explain this, given the larger, more complex genome and increased number of transcription factors.

There is, nevertheless, increased correlation in gene expression for operonic members (Figure 2a,c) and for this reason we analyzed expression patterns of documented operons in *S. coelicolor *and *E. coli*. Using a Z-score normalization procedure (see Materials and methods), gene expression across an operon is normalized to the first gene of the same operon, which allows individual gene expression patterns both within and across operons to be compared. For every position *i *within an operon the distribution of *Z*_*op*,*i *_for operonic genes at position *i *can then be plotted, such that a box in the plots illustrated in Figure 3a-d represents a gene position in a 'virtual' operon. Figure 3a shows a box plot of *Z*_*op*,*i *_values for each gene position in operons in *S. coelicolor*, restricted to five due to lack of experimental data for larger operons. Figure 3a suggests that operons in *S. coelicolor *exhibit 'polar' expression, whereby gene expression generally decreases throughout the operon, with successive genes having lower expression levels than the preceding gene. This does not correspond to the common notion that operonic genes are expressed equally. This apparent downward trend was tested by randomly shuffling gene order in the same operon set using 1,000 simulations, which showed that when the order of the genes in an operon are changed no polarity of expression is observed (data not shown). Using the random (shuffled) *Z*_*op*,*i *_distributions for each position, *p* values were obtained. Although no significance less than *p* < 0.05 was observed for individual positions compared to random, the deviations from expectation for individual genes in given operons is significant, with Z-values exceeding 20 in many instances. We suggest that the downward trend of expression is a characteristic of *S. coelicolor *operons.

**Figure 4:**
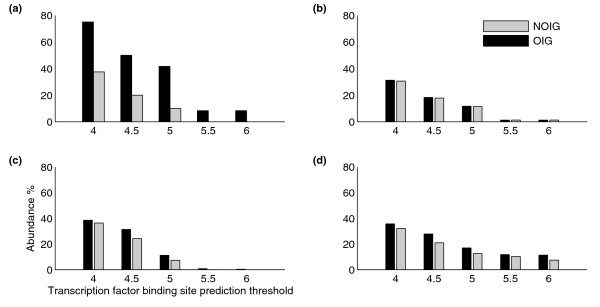
Predicted TFBS abundance in operons. Transcription factor binding site (TFBS) abundance in the upstream intergenic regions of intra-operonic genes with a *Z*_*op*,*i *_greater than μ_*op*,1 _+ σ_*op*,1 _(OIG) and genes with a *Z*_*op*,*i *_less than μ_*op*,1 _- σ_*op*,1 _(NOIG) when using **(a) **the documented *S. coelicolor *operon data set reduced to genes that have an upstream intergenic distance greater than 0, **(b) **a random selection from documented *S. coelicolor *operons, **(c) **Li *et al*.'s [17] TFBS prediction on documented *E. coli *operons, and **(d) **our TFBS prediction on documented *E. coli *operons. NOIG, non-over-represented intra-operonic genes; OIG, over-represented intra-operonic genes.

It is evident that some genes do not follow the trend of downward expression observed in *Streptomyces *operons in Figure 3a. One possible explanation for this increased expression is the presence of internal promoters, a feature that is thought to cause problems in operon prediction methods [6-8,11,14]. Although the prediction of promoter sequences is difficult, TFBSs in prokaryotes tend to be proximal to a promoter [15] and potential internal promoters were assayed by the identification of a putative TFBS. Intra-operonic genes (excluding the initial gene of an operon) were classed as either over-represented intra-operonic genes (OIGs) with a *Z*_*op*,*i *_greater than the μ_*op*,1 _+ σ_*op*,1 _or non-over-represented intra-operonic genes (NOIGs) with a *Z*_*op*,*i *_less than the μ_*op*,1 _- σ_*op*,1_. The abundance of TFBSs within their upstream intergenic regions was studied (Figure 4). Figure 4 shows that there is a consistent over-representation of TFBSs in the OIGS set for *Streptomyces*, not present in randomly selected genes from the same operon set, with a *p* < 0.05 from a chi-square test using TFBS prediction thresholds *m*_*b *_+ *n*σ_*b *_(*n *= 4, 4.5, and 5).

The TFBS prediction algorithm uses position specific weight matrices (PSWMs) to predict likely sites in the upstream regions of candidate genes. Some genes do not possess substantial upstream non-coding sequence, and hence these genes were filtered out in the TFBS tests shown in Figure 4 in order to remove any potential bias. However, the gene *hisB *(SCO2052) is known to have an internal promoter upstream in *E. coli *but has no upstream intergenic sequence in *S. coelicolor*, overlapping the upstream neighboring gene by four bases. This gene was originally assigned to the upregulated set prior to filtering and is, therefore, predicted to be internally promoted, although our approach would not attempt to find a putative TFBS. A substantial proportion of the gene sets fall into this category; 48% of the upregulated data set and 27% of the normal data set had no upstream intergenic sequence. The upregulated genes that fall into this category may well be similar cases in which internal transcription initiation occurs but the internal promoter lies in an intragenic upstream sequence. The significant difference between TFBS abundance for upregulated and normal genes using this method would suggest that TFBS prediction algorithms capable of analyzing overlapping upstream regions should be developed.

There are several reasons why NOIGs have TFBSs identified by our prediction methods: first, it could be that those genes in the majority of cases do not show any upregulation in our restricted experiments but there are conditions when they are upregulated; second, the promoter is unregulated and constitutive activity only enables the gene to reach basal expression [16]; third, a binding site is present and used in termination, a phenomenon found in *Spiroplasma citri *[17]; fourth, experimental error, where expression measurements in the profile are less than the true biological amount; or fifth, due to false positives within our TFBS set, although few false positives are expected at the prediction threshold of *m*_*b *_+ 5σ_*b *_[18].

Only 4 of 55 NOIGs were predicted to have a binding site with a threshold of *m*_*b *_+ 5σ_*b*_; SCO3358 (*cseB*), SCO2610 (*mreC*), SCO5319 (*whiE *protein II), and SCO5625 (*tsf*). No additional information about the transcriptional status of SCO2610 or SCO5319 could be found and, consequently, information for the two remaining genes is briefly discussed here. SCO3358 is the third gene of the *sigE *operon, an operon that has been found to be entirely transcribed only 10% of the time due to termination downstream of the first gene *sigE *[19]. In agreement with this, SCO3358 has reduced expression compared to the first gene of the operon. The binding site we predicted upstream of SCO3358 (*cseB*) may offer an additional route to activate this gene in the operon, as the product of SCO3358 regulates the upstream promoter of the operon [19]. SCO5625 (*tsf*), is the second gene of a bicistronic operon and is expressed less than the first gene (*rpsB*) by a ratio of 2:1 in *S. coelicolor *[13], consistent with the array data presented here. However, the authors of this work [13] could not deduce the likely mechanism and speculated that attenuation, if occurring, may be brought about by a 16 base-pair inverted repeat just upstream of *tsf *in *S. coelicolor*, similar to that found in *E. coli*. Alternatively, a similar attenuation mechanism in *S. coelicolor *to that proposed for the *rpsB*-*tsf *operon of *Spiroplasma citri *may be responsible, where a DNA binding protein interacts with the region immediately downstream of *rpsB *[20]. The binding site found to be bound by a protein just upstream of *tsf *(although how it would influence transcription is not known) by Le Dantec *et al*. [20] was an AT-rich inverted repeat that did not resemble a typical terminator sequence. Interestingly, the inverted repeat predicted by our method to be in the upstream region of *tsf *is also AT-rich. From the 23 OIGs, 5 genes were predicted to have a TFBS (using the threshold of *m*_*b *_+ 5σ_*b*_); SCO2389 (*acpP*), SCO0712 (*lipR*), SCO2390 (*fabF*), SCO4662 (*tuf1*), and SCO5356 (*thrB*). Table 1 details known information about the regulation of these genes, where all but one of the OIGs with predicted binding sites are known to have a promoter in their upstream regions. Although formally classified as a monocistronic operon in *Streptomyces*, *thrB *is included as it is transcriptionally linked to a bicistronic operon involved in the same pathway immediately upstream in *E. coli*, and often part of an operon in other organisms (for example, *B. subtilis*) [21].

The fact that *thrB *was identified as an OIG and a TFBS was also predicted is promising for operon prediction algorithm improvement, since *thrB *is expressed independently in *Streptomyces*. Additionally, *tuf1 *in *Streptomyces ramocissimus *was found to have a TFBS characterized by an inverted repeat upstream of the internal promoter [22], which is speculated to play a role in promoter activation; the promoter sequence, although 'weak' [22], is highly conserved across different strains of streptomycetes, suggesting it plays an important role. Interestingly, the TFBS inverted repeat predicted here in *S. coelicolor *is very similar to the one found in *S. ramocissimus*, suggesting that the mechanism for internal upregulation of *tuf1 *is conserved.

In contrast to the situation observed in *S. coelicolor*, no bias towards increased abundance of TFBSs in OIGs is seen in *E. coli *(Figure 4c,d), again suggesting that there is a general difference in transcriptional regulation between the two species. Of the 65 experimentally validated internal promoter sites (as annotated in RegulonDB [23,24]) within our constructed operon set for *E. coli *(Additional data file 1) 37% of proximal downstream genes (to the promoter) were classed as OIGs by our method using a restricted set of experiments. Of these, 12 genes had upstream intergenic regions that could be tested for TFBSs. Using the TFBS prediction threshold of *m*_*b *_+ 5σ_*b*_, we compared the two sets of PSWMs (see Materials and methods) at finding these known sites. Only 1 site (8%) of the possible 12 was correctly predicted by the matrices built by Li and co-workers [18], whilst our matrices identified 5 (42%). In addition to known sites, our matrices predicted 22 other putative internal control sites in *E. coli *operons (compared to 17 by Li and co-workers matrices [18]), 4 of which are documented in RegulonDB [23,24] as being predicted by computational methods. A complete list of the predicted internal promoters in *E. coli *is given in Additional data file 2.

**Figure 5:**
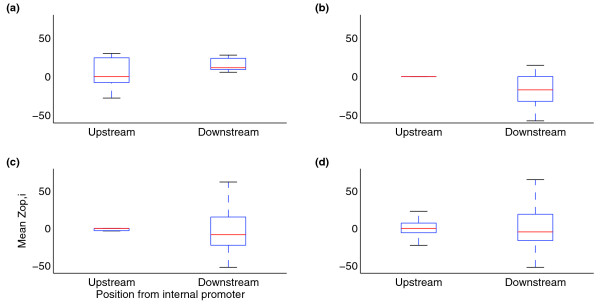
Variation up and down stream of putative internal promoters. Box plots of the mean expression of genes upstream and downstream of the putative internal promoter in **(a) **documented *S. coelicolor *operons using our predictions, **(b) **random simulations of *S. coelicolor *operons, **(c) **documented *E. coli *operons using experimentally known internal promoters, and **(d) **random simulations of *E. coli *operons.

To further test the internal promoter predictions, box plots were made of the mean expression of all the operonic genes upstream and downstream of the putative internal promoter in this restricted set of *S. coelicolor *operons, after normalizing to the first gene of the operon (Figure 5a). In total, 80% of the mean values for genes downstream of the predicted internal promoter have a higher expression compared to the mean values for genes upstream, indicating enhancement of expression due to presence of the promoter (as expected). As a control, when selecting the same number of genes randomly from all of the documented *S. coelicolor *operons and randomly splitting them into upstream and downstream genes (following 1,000 simulations) only 32% were upregulated (Figure 5b). The same trend in *E. coli *is not observed, with higher expression observed downstream of the internal promoter in 57% of cases compared to 49% in equivalent random tests (Figure 5c,d).

**Figure 6:**
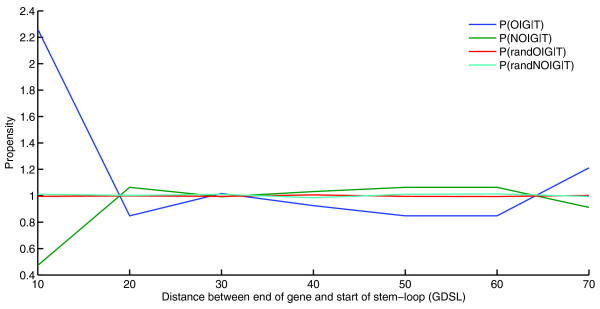
Predicted terminator propensities in operons. Propensity of over-represented intra-operonic genes (OIGs) and non-over-represented intra-operonic genes (NOIGs) predicted to have a terminator in their upstream intergenic region for all GDSL threshold bins compared to random. Propensities were calculated by dividing the fraction of OIGs or NOIGs with a predicted terminator by the fraction of all genes that were OIGs or NOIGs in this restricted 78 gene subset. This was done for non-inclusive 10 base-pair regions moving out from the stop codon of the previous gene. Random values were estimated by random picking from the 78 gene intra-operonic set to represent the OIG and NOIG expressed data sets, repeating 1,000 times.

Internal termination could also play an important role in the control of individual gene expression within operons. Hence, one might expect NOIGs to have a greater abundance of terminators in their upstream regions than OIGs. The prediction of rho-dependent terminators is a difficult task due to the diversity of structures bound by the rho-factor and, therefore, only rho-independent terminators were considered here. Although rho-independent terminator prediction algorithms have been developed by several groups (for example, [25-27]) the %G+C richness of the *S. coelicolor *genome (approximately 72%) and the phylogenetic similarity of *S. coelicolor *and *M. tuberculosis *[12] suggested the use of GeSTer [28]. This algorithm is not dependent on the presence of a U-rich tail following a stem-loop, which might be expected to be absent in *S. coelicolor*. Permitting stem-loops to be sited up to 300 bases downstream of a gene's stop codon, along with GeSTer's default settings [28], resulted in 3,365 predicted terminators. Only about 8% had a U-rich tail, agreeing with expectations. We used the distance between the end of a gene and the start of the stem-loop (GDSL) as a potential metric to reduce false positives, and counted intra-operonic genes from the OIG and NOIG sets with predicted terminators in the upstream intergenic regions. Figure 6 shows the fraction of intra-operonic genes with predicted terminators within the OIG and NOIG sets, excluding the first gene in each operon, which would be expected to have a terminator upstream considering that only 27% of upstream intergenic regions in *S. coelicolor *are divergent.

Regardless of expression status, very few intra-operonic genes are predicted to have an upstream terminator (maximum of about 32% when considering the entire intergenic region). The propensity of genes with predicted terminators for NOIG to OIG are shown in Figure 6, and varies between 0.5 and 2.3. The only clear signal differing from random is observed within 10 bases of the stop codon of the preceding gene, where OIGs have a higher propensity for predicted terminators compared to random, and the opposite effect is seen for NOIGs. Although this appears to be somewhat counterintuitive, we believe this reflects the need to regulate certain genes within an operon independently, which may require the presence of an internal upstream terminator and promoter, as well as a terminator downstream of the gene. This would in effect 'isolate' the gene, allowing its expression independently of the whole operon (where physiological conditions might dictate this). Indeed, we see a similar enrichment for OIGs with a higher propensity for downstream terminators too (1.7 compared to 0.7 for NOIGs). This suggests a more complex mechanism for the regulation and expression of some genes within operons, involving both internal transcriptional initiation and termination.

Finally, to gain some insight into the complexity of intra-operonic gene regulation, intra-operonic genes were split using a TFBS prediction threshold of *m*_*b *_+ 5σ_*b *_and a basic GDSL threshold of 0, to yield four different classes as defined in Table 2: type 1 and type 2 are likely to represent OIGs due to the presence of an upstream TFBS, given what is observed in Figure 4; type 3 are expected to be NOIGs due to the presence of an upstream terminator but the absence of a putative TFBS; and type 4 should represent the majority of genes if there is to be any agreement with the basic operon model as proposed by Jacob and Monod [29]. The proportion of genes falling into each class split into OIGs and NOIGs is also shown in Table 2. Encouragingly, approximately 80% do not have a predicted TFBS or terminator site. Although this is expected to be an overestimate, it is not likely to be a large one given the low false negative rate expected from use of the TFBS matrices (approximately 2%). Furthermore, the majority of type 4 genes are NOIGs and, therefore, have reduced expression compared to the first gene in the operon. This suggests that although internal regulation (promotion and termination) plays an important role in the control of gene expression of genes within some operons, this is by no means a universal mechanism. However, we do expect that some internal TFBSs are missed by current algorithms (about 20% of the genes classed as type 4 are upregulated, although no internal sites could be detected). It is estimated, therefore, that 'internal' gene regulation is likely to operate on 20% to 40% of operonic genes, based on the data currently available.

Interestingly, those genes that have both a TFBS and terminator predicted in their upstream regions (type 1) make up 9% of operonic genes and seem to be mostly upregulated, with a OIG:NOIG ratio of 1.3. This is consistent with the 'isolated' gene expression hypothesis, where both an internal promoter and terminator are required to express this gene independently from the rest of the operon. In addition, there appear to be very few genes in this dataset (3%) that have a TFBS and no terminator (type 2), suggesting that genes that have an upstream TFBS site also have a terminator present, allowing for a tighter control of expression.

## Conclusion

Although the co-regulation of genes in prokaryotes has been shown to be highly conserved in some cases when comparing operonic gene pairs from one organism to the regulon map of another [30], inter-species operon structure is generally not stable [1-4,31]. Instability of operons and, therefore, operonic regulation through the addition, removal, and reorganization of genes during long-term evolution as well as different physiological and developmental states [31] can produce a variety of control mechanisms. Furthermore, it has previously been suggested that patterns of gene co-regulation could be specific to the organism or a set of closely related organisms [32]. Here we provide evidence that demonstrates large differences in the general regulatory mechanisms that *S. coelicolor *and *E. coli *use to control gene expression within operons. This is apparent through the different relative expression patterns across operons and differences in abundance of predicted TFBSs within operons, pointing to different levels of intra-operonic gene regulation between the two species. This raises a note of caution for those attempting to predict operons through the use of expression similarity in other species. Given the dynamistic nature of orthologous genes' operonic organization and differences in their regulation, we cannot expect expression across operons to be similar either.

**Figure 7:**
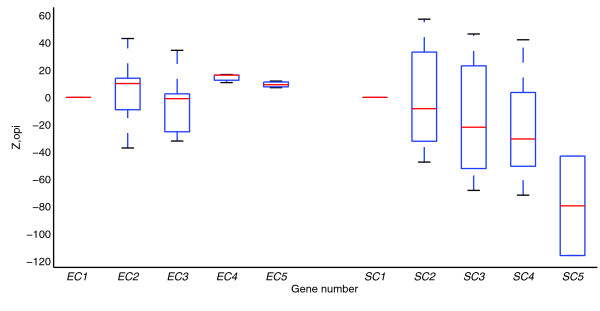
Comparative gene expression for orthologous operons. Boxplot of *Z*_*op*,*i *_scores are shown for orthologous operons, where at least one gene is the same in a documented *S. coelicolor *compared to *E. coli*. Data are shown in *E. coli *(EC) and *S. coelicolor *(SC) using all available experiments.

Operons in *S. coelicolor *exhibit a polarity in expression that is not observed in *E. coli*. When considering the minimum and maximum values obtained for each gene position within an operon (Figure 3), it could also be seen that *E. coli *genes have a tighter distribution of *Z*_*op*,*i *_values than *S. coelicolor*. However, this corresponds to a mixture of functionally equivalent and non-equivalent operon examples. It could be argued that equivalent operons between species that function in a similar response/pathway may have similar regulatory elements and/or expression patterns. An examination of existing comparable data sets suggests otherwise. When comparing documented operons in *E. coli *and the orthologous operons in *S. coelicolor *(defined as containing at least one equivalent gene, yielding a total of 15 pairs matched by gene name), general expression across the operons is different (Figure 7). Using expression data from all available experimental sets, the downward 5' to 3' directionality in expression is retained in *S. coelicolor *operons, but is not shared by orthologous operons in *E. coli*. This has been observed previously when differences in the regulation of orthologous operons across many organisms were characterized [1,30], although not from levels of expression across operons.

The co-transcription of genes in operons allows concerted expression of gene products involved in the same response/pathway [33,34]. However, our data demonstrates a marked polarity of expression in *S. coelicolor *operons. The concept of polarity is not new; as early as 1979, Ullman and colleagues [35] discussed operon polarity as a "salient feature of prokaryotic gene expression, where promoter-distal genes have reduced expression compared to promoter-proximal genes most likely caused by premature termination". This may be caused by a variety of mechanisms, including environmental factors, absence of termination suppressors, and the possible presence of the rho-factor [35]. A further factor is differential mRNA degradation, where genes closer to the 3' end of the polycistronic transcript can be degraded more slowly than genes closer to the 5' end [36].

Although it is not clear from the data presented here which, if any, of these mechanisms is responsible for the differential patterns observed in *S. coelicolor *operons, it is interesting to compare gene expression differences already characterized between *S. coelicolor *and *E. coli*. *S. coelicolor *has an estimated 12.3% of 7,825 genes involved in regulation [12] whilst *E. coli *has 7.2% of 4,829 genes [37], agreeing with the observation that the proportion of genes involved in regulation increases with bacterial genome size [38]. Furthermore, *S. coelicolor *has a large number of sigma factors (65) compared to *E. coli*'s 6. Based on these figures alone we would expect a greater diversity of regulation in *S. coelicolor *than in *E. coli*. A good example of this greater diversity in *S. coelicolor *is the specialization of stress regulons, where each is thought to be controlled by a specific sigma factor or multiple regulatory genes with very few induced proteins being shared between stress responses [12,39]. In contrast, in *E. coli*, sigma 38 (RpoS) brings on a general response to starvation, osmotic, oxidative, and heat stresses [39]. Streptomycetes inhabit highly diverse and dynamic soil and aquatic environments and, as sessile saprophytic organisms, need to constantly modulate gene expression levels to adapt to these changes and modify their metabolism appropriately. This could have led to the development of a more 'punctuated' and flexible transcriptional organization. In this context we also speculate on the potential influence of the high G+C content of *Streptomyces *on the evolution of operons and intra-operon control. The topological consequences of a high G+C (72%) genome might have favored the evolution of 'operons' that can be transcribed segmentally to perhaps modulate torsional stress.

An advantage of operon structure is that, in the majority of cases, all the genes within an operon encode proteins that are needed within the same pathway/response. For *S. coelicolor*, where specialized responses are elicited under certain conditions, specialized promoters within operons would also be an advantage. Indeed, where an increase in expression greater than the expression of the first gene in the operon is needed, we found evidence for a significant abundance of internal promoters in *S. coelicolor*, something not seen in *E. coli*.

Finally, we have shown that 60% to 80% of *S. coelicolor *intra-operonic genes defined in this study did not have any putative internal control sites upstream. Although this represents the majority of the operonic genes, it has serious implications for operon prediction methods. These methods often use the presence of promoters and/or terminators at the start and/or end of an operon as signals to delineate operon boundaries, and clearly in the cases of *S. coelicolor *this is likely to cause mis-prediction of operon membership. The consideration of internal control sites within operons has not yet been implemented in these approaches, and could lead to improvements for some species. Here, we have shown that the use of across-operon expression levels combined with TFBSs and terminator prediction is a strategy capable of allowing for such sites. Work to produce an operon prediction tool that integrates these different sources of information is on-going in our laboratory.

## Materials and methods

### Microarray data

Data from two-color DNA microarrays for *S. coelicolor *were collected from two sources: time-dependent gene expression patterns during development and antibiotic production on solid growth medium, averaged over three replicates stored in ArrayExpress [40,41] (Accession numbers: Experiment, E-MAXD-14; Arrays: A-MAXD-6, UMIST_S COELICOLOR_SC8_7337; A-MAXD-7, UMIST_S COELICOLOR_SC3_6077; A-MAXD-8, UMIST_S COELICOLOR_SC4_6884), resulting in 19 time points with expression ratios extracted from a Genespring^® ^version 5 (Silicon Genetics) output file [42]; and 88 other experiments publicly available in the Stanford Microarray Database (SMD; Additional data file 3) [43,44] with expression ratios calculated from the background-subtracted median values in each channel. In total, 145 publicly available experiments were collected for *E. coli*, using 56 from SMD (Additional data file 4) with expression ratios calculated in the same way as *S. coelicolor *SMD data except for mean intensities being used as medians were unavailable, and taking 89 from the *E. coli *gene expression database of the University of Oklahoma [45-53] (Additional data file 4) with expression ratios calculated from the available control and test values.

### Positive and negative examples of operons

**Figure 8:**
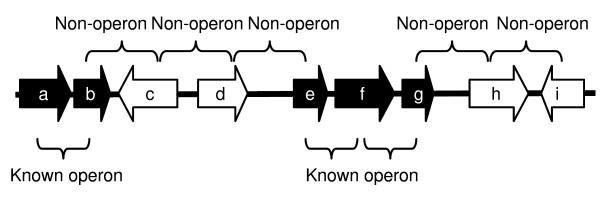
Examples of positive and control sets of operons/non-operons.

Operon definitions were based on the annotated genome of *E. coli *K12 strain MG1655 from GenBank [54,55] [GenBank:NC_000913] and the *S. coelicolor *chromosome (not cosmid) in EMBL [56,57] [EMBL:AL645882 version 2]. Positive examples of operons were collected through searching the literature for operons in *S. coelicolor *(Additional data file 5) and from the transcriptional unit annotation in EcoCyc for *E. coli *[58,59] (Additional data file 6). Negative examples of operons were collected from knowledge of basic operon structure applicable to both organisms (Figure 8). Assuming that the entire polycistronic transcript is documented, non-operons of length 2 were formed by using the initial gene of the operon and its upstream neighboring gene if that neighbor is transcribed in the same direction (for example, gene pair d-e), and the last gene in the documented operon and its downstream neighboring gene, again if it is transcribed in the same direction (for example, gene pair g-h). To increase the size of the non-operonic data set a non-operon of length 3 was also formed by collecting triplets of genes that are transcribed in the opposite direction (for example, genes b-c-d). In total, 35 operons and 1,282 non-operons were collected for *S. coelicolor*, and 325 operons and 821 non-operons were collected for *E. coli*.

### TFBS prediction

Lists of putative TFBSs for both *S. coelicolor *and *E. coli *were collected by searching upstream intergenic regions for over-represented dimers using the method of Li *et al*. [18], searching against the same *E. coli *K12 strain MG1655 from GenBank [54,55] and *S. coelicolor *chromosome from EMBL [56,57]. This method defines putative TFBSs as PSWMs for each putative site, based on the statistical over-representation of dimeric words (dyads) in defined sequence sets. Our implementation of this method yielded slightly different results from the published ones; however, we used both sets of TFBSs for *E. coli *- the published TFBSs of Li *et al*. [18], giving 849 putative sites, and the TFBSs collected with our implementation of Li *et al*.'s method [18], resulting in 1,506 putative sites. Comparisons between the two data sets were performed and we were able to find all of those published by Li *et al*. [18] plus additional sites and we therefore use this as an alternative set. For *S. coelicolor*, a TFBS list was built by searching upstream of all genes on the chromosome and applying the same method [60], resulting in 3,628 putative sites defined by PSWMs. Here we define 'upstream' as the maximum of 300 nucleotides or the intergenic distance to the stop codon of the previous gene. Previous work applying a similar approach on *S. coelicolor *[60] reported 2,497 putative TFBSs. However, using dyad word lengths of 3 to 5 nucleotides instead of 4 nucleotides results in additional matrices, as well as all of the matrices found previously [60].

Hence, for each putative TFBS the defining sequences can be matched back against the PSWM, resulting in a mean score *m*_*d *_with standard deviation σ_*d*_. Similarly, a mean score *m*_*b *_with standard deviation σ_*b *_is obtained matching the PSWM against background sequences (all upstream sequences) as described in Li *et al*. [18]. For a sequence to be predicted to contain a given TFBS, the score of the sequence against the PSWM must be higher than *m*_*d *_- 2σ_*d *_and *m*_*b *_+ *x*σ_*b*_, where *x *is used to represent trials of different thresholds as discussed later in this report; since this is the only part of the threshold that changes, the TFBS prediction threshold used will be represented by *m*_*b *_+ *x*σ_*b*_.

### Normalization of microarray data

A per-chip normalization strategy was used throughout. This was found to be the best normalization strategy with regards to operon prediction, since the general trajectory of expression is retained. Hence, all log_2 _expression ratios for each experiment in the microarray data sets collected for *S. coelicolor *and *E. coli *were calculated as follows:



We define the expression level of each gene in a given operon of *m *genes as *g*_*i *_for *i *= 1, *m*. As the first gene of an operon (*g*_1_) is the first to be transcribed it is expected that the transcription level of the downstream genes should be equal to the expression of *g*_1 _(given the expectation that they are co-transcribed in a single polycistronic unit) and, therefore, the expression level of *g*_1 _is taken as a representative of the operon's expression level (μ_*op*,1_). Using all available experiments (*j*) μ_*op*,1 _is calculated by:



Where *n *= total number of experiments (107 for *S. coelicolor *and 145 for *E. coli*). μ_*op*,1 _has a standard deviation associated with it (σ_*op*,1_):



Each gene in an operon (*g*_*i*_) can then be expressed as a Z-score, normalizing its value of expression compared to the μ_*op*,1_, *Z*_*op*,*i *_can then be calculated:



The Z-score provides a simple metric that measures the expression level of genes in an operon with respect to the first, with an expectation that genes should have small Z-scores close to 0 if the measured expression of every gene in an operon is truly uniform. In addition, this procedure facilitates cross-operon comparison by normalizing the expression of the first gene in every operon to 0.

## Additional data files

The following additional data are available with the online version of this paper. Additional data file 1 lists 65 *E. coli *genes found in our constructed data set of operons that have an experimentally validated upstream internal promoter. Additional data file 2 comprises two tables: the top table describes OIGs in *E. coli *found to have a putative TFBS site using our own PSWMs, their *Z*_*op*,*i *_score (in comparison to the first gene of the respective operon) and whether they are experimentally known (or computationally predicted); the bottom table describes *E. coli *OIGs predicted to have an upstream internal promoter using Li *et al*. [18] matrices. Additional data file 3 lists the SMD Experiment IDs used for *S. coelicolor*. Additional data file 4 lists the Oklahoma database experiment IDs and SMD Experiment IDs used for *E. coli*. Additional data file 5 documents experimentally validated operons used in this analysis for *S. coelicolor*. Additional data file 6 documents experimentally validated operons used in this analysis for *E. coli*.

## Supplementary Material

Additional File 1 Sixty-five *E. coli *genes found in our constructed data set of operons that have an experimentally validated upstream internal promoter. Click here for file

Additional File 2 The top table describes OIGs in *E. coli *found to have a putative TFBS site using our own PSWMs, their Z_*op*,*i *_score (in comparison to the first gene of the respective operon) and whether they are experimentally known (or computationally predicted); the bottom table describes *E. coli *OIGs predicted to have an upstream internal promoter using Li *et al*. [18] matrices. Click here for file

Additional File 3 SMD Experiment IDs used for *S. coelicolor*. Click here for file

Additional File 4 Oklahoma database experiment IDs and SMD Experiment IDs used for *E. coli*. Click here for file

Additional File 5 Experimentally validated operons used in this analysis for *S. coelicolor*. Click here for file

Additional File 6 Experimentally validated operons used in this analysis for *E. coli*. Click here for file

## Figures and Tables

**Table 1:** Over-represented intra-operonic genes with predicted TFBS*

Gene name	Known internal promoter?	Organism (reference if known)
SCO2389 (*acpP*)	Yes	*Escherichia coli *[61]
SCO0712 (*lipR*)	?	-
SCO2390 (*fabF*)	Yes	*Escherichia coli *[61]
SCO4662 (*tuf1*)	Yes	*Streptomyces ramocissimus *[22]
SCO5356 (*thrB*)	Monocistronic	*Streptomyces *[21]

*OIGs that have a TFBS predicted using threshold background mean + (5 × background standard deviation) and whether they are known to have an internal promoter and in which organism this has been found in.

**Table 2:** Classification of genes by predicted regulatory sites and fraction of OIGs and NOIGs that fall into each gene type

Type number	Predicted TFBS?	Predicted terminator?	OIG (%)	NOIG (%)
1	Y	Y	5.1	3.9
2	Y	N	1.3	1.3
3	N	Y	2.6	7.8
4	N	N	20.5	57.8
